# Screening for Coronavirus Disease 2019 (COVID-19) at the Pediatric Emergency Department During Different Pandemic Phases

**DOI:** 10.3389/fped.2021.749641

**Published:** 2021-11-05

**Authors:** Indrė Stacevičienė, Sigita Burokienė, Aušra Steponavičienė, Daiva Vaičiūnienė, Roma Puronaitė, Augustina Jankauskienė

**Affiliations:** Institute of Clinical Medicine, Faculty of Medicine, Vilnius University, Vilnius, Lithuania

**Keywords:** COVID-19, SARS-CoV-2, children, screening, PCR, pediatric emergency department

## Abstract

The wide spectrum of COVID-19 symptoms complicates the selection of target groups for screening. We aimed to compare data of children screened for COVID-19 at the pediatric emergency department in Vilnius between different phases throughout 1 year (Phase I: March–May, 2020; Phase II: June–September, 2020; and Phase III: October, 2020–February, 2021) and to evaluate the possible predictors of the disease. SARS-CoV-2 PCR tests were positive for 2.7% of tested children (248/9,238), significantly higher during the Phase III (5.5%) compared with the Phase I (0.6%, *p* = 0.000) and Phase II (0.3%, *p* = 0.000). Infants and teenagers (12–17 years) accounted for a larger proportion of COVID-19 patients (24.6 and 26.2%, respectively) compared to other age groups: 1–2 years (18.9%), 3–6 years (14.9%), and 7–11 years (15.3%). There were more COVID-19 cases among children with a known SARS-CoV-2 exposure compared to those who did not declare any contact (18.2 vs. 1.1%, *p* = 0000). When symptoms were adjusted for age, gender and known exposure to SARS-CoV-2, we found that fever (OR 2.66; 95% CI 1.89–3.81), pharyngitis (OR 1.35; 95% CI 1.01–1.80), headache (OR 1.81; 95% CI 1.09–2.90), and anosmia/ageusia (OR 6.47; 95% CI 1.61–22.47) were the most significant predictors.

**Conclusion:** Although high numbers of testing were maintained throughout the year, the positive test results were significantly higher during the Phase III. Age (<1 year, 12–17 years), a history of exposure to SARS-CoV-2 and some symptoms, such as fever, pharyngitis, headache and anosmia/ageusia could aid in targeting groups for screening for COVID-19 in children.

## Introduction

The global spread of severe acute respiratory syndrome coronavirus 2 (SARS-CoV-2) infection caused more than 110 million cases with over 2.4 million deaths worldwide between late December 2019 and February 2021 (1). During this approximate one-year period almost two hundred thousand (200,000) cases of the coronavirus disease 2019 (COVID-19) were diagnosed in Lithuania, including over fourteen thousand (14,000) children. Vilnius was a leading region in confirmed pediatric cases (32.4%, *n* = 4,643). There were over 3,000 deaths related to COVID-19 among adults and no deaths among children in Lithuania (2).

In studies performed to date, most children had mild or moderate disease (3, 4). Some had severe cases, including fatalities (5, 6). The symptoms of acute COVID-19 infection usually mimic other childhood illnesses, primarily respiratory or gastrointestinal infections (7). Pediatric Inflammatory Multisystem Syndrome (PIMS), following COVID-19, must also be differentiated from various bacterial and viral infections as well as Kawasaki disease (8). This lack of specificity of signs or symptoms makes screening for identification of SARS-CoV-2 infection in children particularly challenging.

Several COVID-19 management strategies were followed in Lithuania during the year: quarantine (March–May 2020); eased restrictions (June–September 2020); and, as a more threatening second wave began (October 2020), a second lock-down (November 2020–February 2021). The aim of our study was to compare data of children screened for COVID-19 during this time and to evaluate the possible predictors of the disease.

## Materials and Methods

Patients were recruited retrospectively and prospectively in a single center, the Vilnius University Hospital Santaros Klinikos, from March 1, 2020 to February 28, 2021. The study was approved by the Vilnius Regional Biomedical Research Ethics Committee (No. 2020/8-1269-737). Children were screened for SARS-CoV-2 according to the recommendations of the Ministry of Health, which changed several times according to the epidemiological situation of COVID-19 in the country.

In Phase I, at the onset of the pandemic, patients were screened who presented with acute respiratory infection (sudden onset with at least one of the following: fever, cough, shortness of breath) and one of the following: travel within 14 days of the onset of symptoms to areas with presumed ongoing community transmission of COVID-19 (different countries); close contact with a confirmed or suspected case of COVID-19 infection; and no other reason for the onset of symptoms. In addition, beginning in May, all children were routinely tested for SARS-CoV-2 before inpatient admission. The same testing strategy was used in Phases II and III. Beginning in February 2021, testing was no longer required for patients with previous COVID-19 infection, confirmed by SARS-CoV-2 PCR or SARS-CoV-2 antigen testing <90 days prior to hospital admission, or for patients who tested positive for a serological antibody test <60 days before admission.

Patients under 18 years who were tested for COVID-19 at the pediatric emergency department (PED) were enrolled in the study. Patients were either symptomatic for COVID-19 or were admitted to the hospital related to chronic diseases. Nasopharyngeal swabs were taken and real-time reverse-transcriptase PCR tests were performed for SARS-CoV-2. The test samples were taken by several trained doctors.

Basic characteristics (age, gender), source of infection, clinical symptoms, laboratory results and outcome data (hospitalized/discharged, recovered/deceased) were obtained from patients electronic medical records. COVID-19 was diagnosed by a positive PCR test, while other final diagnoses were taken from routine doctors' notes without specified criteria.

**Figure 1:**
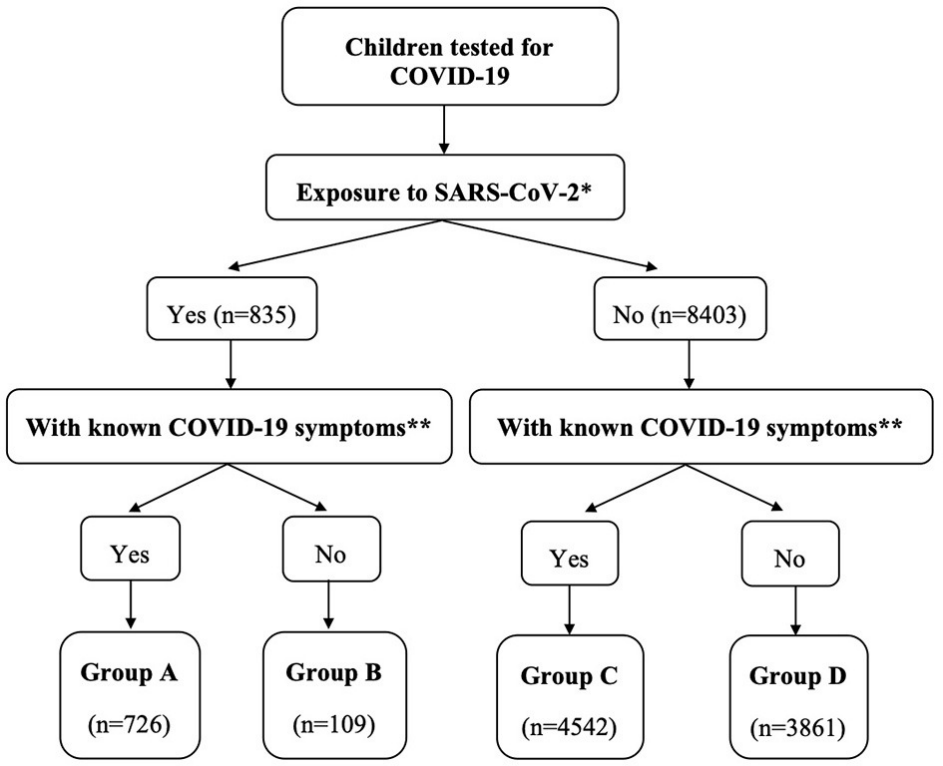
Stratification of enrolled children.

We stratified the patients into four groups (A–D) according to known exposure to SARS-CoV-2 and clinical symptoms: Group A, known exposure with known symptoms; Group B, known exposure with no known symptoms; Group C, no known exposure and known symptoms; and Group D, no known exposure and no known symptoms (Figure 1). We distinguished three periods of time, based on the number of COVID-19 cases in the community (2): Phase I: quarantine during the first wave (March–May 2020); Phase II, eased restrictions as cases decreased (June–September 2020); and Phase III, the second wave (October 2020–February 2021). We compared data within these three time periods, analyzed predictors of COVID-19 in children and the most common diagnoses among symptomatic patients that can mimic SARS-CoV-2 infection. In addition, we looked for the possible cases of PIMS and the outcome of COVID-19.

Statistical analyses were performed with R version 4.1.0. Categorical data were presented as frequencies and percentages and analyzed using Pearson's chi-square or Fisher's exact test where appropriate. For continuous data, medians/interquartile range (IQR) and ranges were calculated. To compare the proportions of positive test results in the four groups (A, B, C, D) during our three periods of the COVID-19 pandemic, a Cochran–Mantel–Haenszel (CMH) test was used. Univariable and multivariable logistic regression models were used to identify symptoms associated with positive SARS-CoV-2 test results. Univariable logistic regression analyses were first performed for all independent variables, with positive SARS-CoV-2 tests. Multivariable logistic regression models were constructed for all independent variables, adjusted for age, gender and known exposure to SARS-CoV-2. Both crude and adjusted odds ratios with 95% confidence intervals were calculated when fitting the logistic regression model. A *p* < 0.05 considered as statistically significant.

## Results

**Figure 2:**
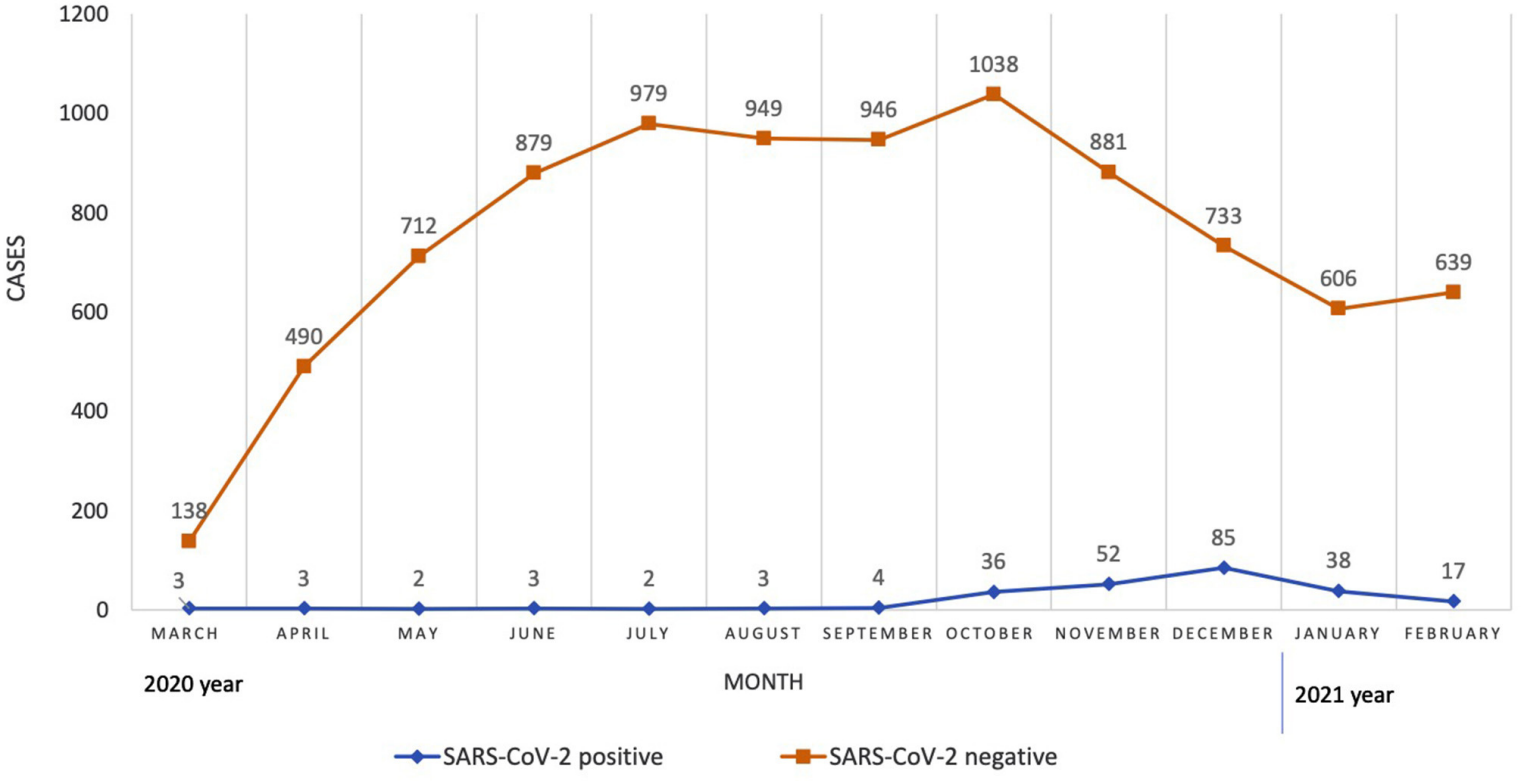
Distribution of SARS-CoV-2-positive and SARS-CoV-2-negative cases on a monthly basis.

During the first year of the COVID-19 pandemic in Lithuania, 9,238 children were screened for SARS-CoV-2 at the pediatric emergency department in Vilnius. The median age of those who were tested was 4.0 years (IQR 1.0–10.0, range 0–17 years). The gender ratio was 1.14 males to females. SARS-CoV-2 PCR tests were positive for 2.7% of children (248/9,238). There were significantly more COVID-19 cases during the Phase III (5.5%, 228/4,125) compared with Phase I (0.6%, 8/1,348; *p* = 0.000) and Phase II (0.3%, 12/3,765; *p* = 0.000). Distribution of SARS-CoV-2-positive and SARS-CoV-two-negative cases on a monthly basis is shown in Figure 2.

There were no significant gender differences: 2.8% of girls (120/4,316) and 2.6% of boys (128/4,922) had positive PCR test results. The median age of SARS-CoV-2-positive patients was 4.0 years (IQR 1.0–12.0, range 0–17 years). Infants (<1 year) and teenagers (12–17 years) accounted for a larger proportion of COVID-19 patients (24.6 and 26.2%, respectively) compared to other age groups: 1–2 years (18.9%, *p* = 0.000 and *p* = 0.001), 3–6 years (14.9%, *p* = 0.000 and *p* = 0.000) and 7–11 years (15.3%, *p* = 0.002 and *p* = 0.037).

There were more COVID-19 cases among children with a known COVID-19 exposure compared to those who did not declare any contact (18.2%, 152/835 vs. 1.1%, 96/8,403, *p* = 0000). Similarly, there were more COVID-19 cases among symptomatic children compared to those who were asymptomatic (3.7%, 209/5,268 vs. 1%, 39/3,970, *p* = 0000).

The most common symptoms of COVID-19 patients were fever (80.6%, 200/248), pharyngitis (58.1%, 144/248), cough (27%, 67/248) and rhinorrhoea (21.8%, 54/248). Shortness of breath and anosmia/ageusia were rare, 4% (10/248) and 2% (5/248), respectively. Gastrointestinal symptoms were seen for 13% of COVID-19 patients: vomiting (12.9%, 32/248), abdominal pain (13.3%, 33/248) and diarrhea (8.9%, 22/248). Other symptoms included headache (10.1%, 25/248), weakness/fatigue (5.2%, 13/248), rash (3.6%, 9/248), myalgia (0.8%, 2/248) and seizures (0.4%, 1/248). A significant proportion of COVID-19 patients (15.8% (39/248)) were asymptomatic.

Most of the tested children (90.9%) fell into groups C and D (49.1 and 41.8%, respectively). The largest groups contained the smallest number of COVID-19-positive cases (1.5%, 69/4,542 in Group C and 0.7%, 27/3,861 in Group D). There were significantly more COVID-19-positive patients in groups A and B (19.3%, 140/726 and 11.0%, 12/109, respectively), compared to groups C and D (p=0.000). Moreover, group A contained significantly more COVID-19 cases than group B (*p* = 0.037).

Characteristics of SARS-CoV-2-positive subjects during Phases I–III are shown in Table 1. There were more COVID-19 children with a known contact during Phases I and II compared with Phases III (*p* = 0.034). No significant differences between periods were found when the patients were compared by gender, age groups or known COVID-19 symptoms. The distribution of groups A–D among all tested children during the different periods looks similar, while distribution among COVID-19-positive patients is different (*p* < 0.001). In Phase I, all COVID-19 patients except one were in group A. During Phases II and III, COVID-19 patients were found in all four groups, with a predominance in group A. There were more COVID-19 patients from the group B during Phase II and more COVID-19 patients from group C during Phase III (Supplementary Figure 1).

**Table 1:** Comparison of SARS-CoV-2 positive subjects in Phases I–III.

**Characteristics**	**Phase I, *n* = 8**	**Phase II, *n* = 12**	**Phase III, *n* = 228**	**Total, *n* = 248**
	**(2020.03–2020.05)**	**(2020.06–2020.09)**	**(2020.10–2021.02)**	**(2020.03–2021.02)**
Male	3 (37.5)	8 (66.7)	117 (51.3)	128 (51.6)
Female	5 (62.5)	4 (33.3)	111 (48.7)	120 (48.4)
Median age (IQR)	4.0 (0.5–11,25)	11.0 (3.0–16.0)	3.0 (0.25–12.0)	4.0 (1.0–12.0)
<1 year	2 (25)	2 (16.7)	57 (25)	61 (24.6)
1–2 years	1 (12.5)	0 (0)	46 (20.2)	47 (19)
3–6 years	2 (25)	2 (16.7)	33 (14.5)	37 (14.9)
7–11 years	1 (12.5)	3 (25)	34 (14.9)	38 (15.3)
12–17 years	2 (25)	5 (41.7)	58 (25.4)	65 (26.2)
With known exposure^*^	8 (100)	9 (75)	135 (59.2)	152 (61.3)
Without known exposure	0 (0)	3 (25)	93 (40.8)	96 (38.7)
With known COVID-19 symptoms^**^	7 (87.5)	7 (58.3)	195 (85.5)	209 (84.3)
Without known COVID-19 symptoms	1 (12.5)	5 (41.7)	33 (14.5)	39 (15.7)

* *A history of traveling to affected areas during the last 2 weeks or a close contact with a confirmed or probable COVID-19 case in the last 14 days*.

** *A sudden onset of at least one of the following: cough, fever, shortness of breath*.

Univariable logistic regression analyses showed that age (<1 year), known exposure to SARS-CoV-2, fever, cough, rhinorrhea, pharyngitis, headache, weakness/fatigue, anosmia/ageusia were associated with a positive SARS-CoV-2 test. When symptoms were adjusted for age, gender and known exposure to SARS-CoV-2, we found that fever, pharyngitis, headache and anosmia/ageusia were the most significant predictors (Table 2).

**Table 2:** Predictors of positive SARS-CoV-2 test results.

**Characteristic**	**Unadjusted**			Adjusted^*^		
	**OR**	**OR 95% CI**	***p*-value**	**OR**	**OR 95% CI**	***p*-value**
Gender (Ref. female)						
Male	0.93	0.73–1.20	0.594	-	-	-
Age group (Ref. < 1 year)						
1–2 years	**0.44**	**0.29**–**0.64**	**<0.001**	-	-	-
3–6 years	**0.40**	**0.26**–**0.60**	**<0.001**	-	-	-
7–11 years	**0.53**	**0.35**–**0.80**	**0.003**	-	-	-
12–17 years	0.82	0.82–1.18	0.280	-	-	-
Known exposure to SARS-CoV-2 (Ref. no)						
Yes	**19.26**	**14.77**–**25.22**	**<0.001**	-	-	-
Fever > 37.3°C (Ref. no)						
Yes	**3.63**	**2.67**–**5.05**	**<0.001**	**2.66**	**1.89**–**3.81**	**<0.001**
Cough (Ref. no)						
Yes	**2.81**	**2.09**–**3.72**	**<0.001**	1.36	0.97–1.87	0.067
Shortness of breath (Ref. no)						
Yes	1.27	0.62–2.29	0.467	0.92	0.44–1.76	0.823
Rhinorrhoea (Ref. no)						
Yes	**2.02**	**1.47**–**2.73**	**<0.001**	1.05	0.73–1.48	0.787
Pharyngitis (sore throat/ pharyngeal erythema; Ref. no)						
Yes	**2.01**	**1.56**–**2.61**	**<0.001**	**1.35**	**1.01**–**1.80**	**0.046**
Gastrointestinal symptoms (Ref. no)						
Yes	1.08	0.807–1.42	0.613	0.96	0.73–1.25	0.789
Rash (Ref. no)
Yes	0.63	0.30–1.16	0.176	0.69	0.32–1.31	0.292
Headache (Ref. no)
Yes	**2.34**	**1.49**–**3.51**	**<0.001**	**1.81**	**1.09**–**2.90**	**0.018**
Myalgia (Ref. no)						
Yes	2.21	0.36–7.31	0.279	1.17	0.17–4.82	0.846
Weakness/Fatigue (Ref. no)						
Yes	**2.16**	**1.16**–**3.69**	**0.008**	1.38	0.69–2.54	0.331
Anosmia/ageusia (Ref. no)						
Yes	**13.19**	**4.24**–**34.8**	**<0.001**	**6.47**	**1.61**–**22.47**	**0.005**
Seizures (Ref. no)						
Yes	0.20	0.01–0.89	0.107	0.24	0.01–1.13	0.162

* *Adjusted by age, gender, known exposure to SARS-CoV-2*.

Upper respiratory tract infection was diagnosed for 16.3% (858/5,268) of symptomatic children; lower respiratory tract infection for 3.9% (204/5,268); infectious gastroenteritis and/or colitis for 9.6% (702/5,268); unspecified viral infection for 15.5% (814/5,268); unspecified bacterial infection for 3.1% (162/5,268) cases. The comparison of the most common diagnoses during different periods is shown in Supplementary Figure 2. During Phase I, a majority (80%, 348/433) of children with known COVID-19 symptoms were also tested for influenza A and B viruses. Eight children were diagnosed with flu: four children with influenza A, the other four with influenza B. Each of these had a negative SARS-CoV-2 PCR test. During Phases II and III, 48 and 39 children were tested, respectively, and none were diagnosed with influenza A or B. PIMS was diagnosed in six tested children, all during Phase III. Only one patient with PIMS was SARS-CoV-2-positive.

COVID-19 disease was mild to moderate (34.3%, 85/248, and 72.9%, 156/248, respectively). Only 7 cases (0.9%) were severe. About one-third of COVID-19 patients were hospitalized (31%, 77/248). All COVID-19 patients recovered. No patients died.

## Discussion

Our study is one of the few that compares COVID-19 data in children during different phases of a pandemic. We tested almost 10,000 children of different age groups during a one-year period at the hospital emergency department. This was possible because of Lithuania's extensive country-wide testing program. Although high numbers of testing were maintained throughout the year and the distribution of A–D groups among all tested children was similar, positive test results were significantly higher in Phase III. Similarly, cases of COVID-19 in children increased throughout the country, beginning with 56 and 288 cases during Phases I and II, with a sudden increase to fourteen thousand three (14,003) cases in Phase III. Children accounted for 3.5, 8.7, and 7.2% of all COVID-19 cases in Lithuania in Phases I–III, respectively (2). The numbers of COVID-19-positive children are increasing both in European countries (9) and the USA. The latter has seen an increase in lab-confirmed COVID-19 cases in children from 1.7% in February–April, 2020 to 13.1% in February, 2021 (5, 10).

Our early low numbers may have resulted from low circulation of COVID-19 in Lithuania (2) due to the major social contact restrictions imposed, the first lockdown in the country. Despite the restrictions being eased in Phase II, the amount of testing doubled in the hospital and the number of confirmed COVID-19 cases was minimal. One possible explanation is that this was due to the warm weather season, as most studies show an inverse relationship between outdoor temperature and the transmission of SARS-CoV-2 (11). The second quarantine that began in November was not as restrictive as the first. Day care centers were open, the circulation of COVID-19 was much higher in the country (2) and there were more COVID-19 cases among children tested at the ED.

Infants (<1 year) and teenagers (12–17 years) accounted for a larger proportion of COVID-19 patients in our study. According to CDC, COVID-19 incidence among adolescents aged 12–17 years was ~ twice that in children aged 5–11 years (12). A review of household and community transmission showed that susceptibility to infection for children younger than 10 years is estimated to be significantly lower (13). In a multicenter European study, in which our hospital also took part in April 2020, about two-thirds of laboratory-confirmed SARS-CoV-2 patients were among participants younger than 12 months (29%) and older than 10 years (34%; up to 18 years) (3). Another multicenter Italian study found that hospital admission was inversely related to age among infected children (14). Thus, in the studies like ours where data are captured from children who were seen or managed within the hospital setting, adolescents, and infants seem more vulnerable to COVID-19 infection compared to other age groups. This could be explained by adolescents having more contacts because of their higher socialization rate, while the high rate among infants could be explained by the tendency of parents of infants to promptly seek medical attention for their children even when symptoms are mild.

Patient history of exposure to SARS-CoV-2 is of great importance. A high difference in COVID-19 cases was found among children with a known COVID-19 exposure compared to those who had no known contact (18.2 vs. 1.1%). This difference was significantly greater than the difference between symptomatic and asymptomatic children (3.7 vs. 1%). At the start of the pandemic all COVID-19 cases had a contact with a confirmed COVID-19 family member. Later, as the number of cases increased, it became difficult to identify all known contacts and a large proportion of COVID-19 patients (40.8%) had no known any contact in Phase III. This makes screening and identifying target groups difficult. For this reason, contact identification and tracking should remain one of the highest priorities in COVID-19 pandemic control.

As the majority (91%) of tested children were without known exposure to SARS-CoV-2, it was important to search for other predictors of positive SARS-CoV-2 test results. We observed a wide range of non-specific symptoms at presentation. Most of our COVID-19 patients (84.3%) had at least one of the symptoms, included in ECDC clinical criteria: fever, cough, shortness of breath or anosmia/ageusia (15). When symptoms were adjusted for age, gender and known exposure to SARS-CoV-2, we found that fever, pharyngitis, headache and anosmia/ageusia were the most significant predictors. All of these symptoms have been reported in other countries with fever and cough being the most predominant (4, 16). Different than other studies, we found sore throat and headache but not cough as predictors, however clinical significance of an odds ratio for pharyngitis is weak. The ability to elicit symptoms is naturally limited in pediatric medicine, especially among infants and young children. The absence of specific symptoms complicates the selection of target groups for screening. Routine testing for SARS-CoV-2 is therefore important due to the difficulty in diagnosing COVID-19 in children based only on their symptoms. Moreover, some children present without any symptoms (11, 15). The apparent need to test children without known COVID-19 symptoms must be evaluated in the light of cost-effectiveness.

Our data for COVID-19 patients during Phases I and II should be analyzed with caution because of low number of cases, 8, and 12, respectively. During Phase III, when we had a higher number (*n* = 228) of SARS-CoV-2 positive patients, 14.5% were asymptomatic. This finding is consistent with other studies, in which 16–26% of children with COVID-19 were asymptomatic (4, 16). It is possible that widespread screening would reveal a higher percentage of COVID-19-positive children among those who are asymptomatic. We also confirm findings from previous studies (3, 4, 16–18) that the majority of COVID-19 cases in children is mild or moderate, without lethal outcome.

The most common diagnoses among symptomatic patients that can mimic SARS-CoV-2 were upper respiratory tract infections, infectious gastroenteritis or colitis and other viral infections. During Phase I, a majority (80%) of children with known COVID-19 symptoms were also tested for influenza A and B viruses with only eight cases (2%) diagnosed with flu. During Phase III we found no cases of flu. This could be due to a small number of children tested, however, nation-wide there were 53 times fewer cases of flu (*n* = 302) compared with the 2019–2020 flu season (*n* = 16 218) and 164 times fewer compared with the 2018–2019 flu season (*n* = 49 677) (19). The last flu season (2020 autumn/2021 spring) throughout Europe was most unusual because of inexplicably low numbers of flu cases comparing with the previous years (20).

The strength of our study is in the high number of children of various ages tested for COVID-19 with epidemiological and clinical data over the course of a year over different periods of time. However, there are limitations. The information presented in our study is taken from hospital-standardized electronic admission records, not tailored specifically. Thus, the data may be subject to inaccuracies and incomplete reporting, as the entries were made by many specialists. In addition, the statistical comparison between the periods was limited due to the low number of positive COVID-19 cases at the onset of the pandemic, but this just reflects the real epidemiological situation and emphasizes the differences.

Finally, we conclude that screening for COVID-19 in children is exceptionally challenging due to the diverse and non-specific symptoms of infection they present. Age (<1 year, 12–17 years), a history of exposure to SARS-CoV-2 and certain symptoms, such as fever, pharyngitis, headache and anosmia/ageusia could help to select the target groups for screening for COVID-19 in children.

## Data Availability Statement

The original contributions presented in the study are included in the article/Supplementary Material, further inquiries can be directed to the corresponding author/s.

## Ethics Statement

The studies involving human participants were reviewed and approved by Vilnius Regional Biomedical Research Ethics Committee. Written informed consent from the participants' legal guardian/next of kin was not required to participate in this study in accordance with the national legislation and the institutional requirements.

## Author Contributions

IS: designed the study, prepared all the study documents, analyzed patient data, performed the calculations and statistical analysis, performed the literature review and took the lead in writing the manuscript, and designing the figures. SB: was a leading investigator, conceived, initiated and designed the study, contributed to data interpretation, and revised it critically. AS: analyzed patient data and performed the calculations. DV and AJ: conceived, initiated and designed the study, contributed to data interpretation, and revised it critically. RP: performed the calculations and statistical analysis. All authors have read and approved the manuscript in its current state.

## Conflict of Interest

The authors declare that the research was conducted in the absence of any commercial or financial relationships that could be construed as a potential conflict of interest.

## Publisher's Note

All claims expressed in this article are solely those of the authors and do not necessarily represent those of their affiliated organizations, or those of the publisher, the editors and the reviewers. Any product that may be evaluated in this article, or claim that may be made by its manufacturer, is not guaranteed or endorsed by the publisher.

## References

[1] World Health Organization. Weekly Epidemiological Update - 23 February 2021. Available online at: https://www.who.int/publications/m/item/weekly-epidemiological-update-23-february-2021 (accessed June 18, 2021).

[2] Oficialiosios statistikos portalas. COVID-19 Statistics of the Previous Day. Available online at: https://osp.stat.gov.lt/praejusios-paros-covid-19-statistika (accessed June 18, 2021).

[3] Götzinger F Santiago-García B Noguera-Julián A Lanaspa M Lancella L Calò Carducci FI. COVID-19 in children and adolescents in Europe: a multinational, multicentre cohort study. Lancet Child Adolesc Health. (2020) 4:653–61. 10.1016/S2352-4642(20)30177-2 32593339 PMC7316447

[4] Parcha V Booker KS Kalra R Kuranz S Berra L Arora G. A retrospective cohort study of 12,306 pediatric COVID-19 patients in the United States. Sci Rep. (2021) 11:10231. 10.1038/s41598-021-89553-1 33986390 PMC8119690

[5] American Academy of Pediatrics and the Children's Hospital Association. Children and COVID-19 State Data Report 4.29.21 FINAL.pdf. Available online at: https://downloads.aap.org/AAP/PDF/AAP%20and%20CHA%20-%20Children%20and%20COVID-19%20State%20Data%20Report%204.29.21%20FINAL.pdf (Accessed Jun 18, 2021)

[6] Bixler D. SARS-CoV-2–associated deaths among persons aged 21 Years — United States, February 12–July 31, 2020. MMWR Morb Mortal Wkly Rep. (2020) 69:1324−9. 10.15585/mmwr.mm6937e4 32941417

[7] Zimmermann P Curtis N. Coronavirus infections in children including COVID-19: an overview of the epidemiology, clinical features, diagnosis, treatment and prevention options in children. Pediatr Infect Dis J. (2020) 39:355–68. 10.1097/INF.0000000000002660 32310621 PMC7158880

[8] Whittaker E Bamford A Kenny J Kaforou M Jones CE Shah P. Clinical characteristics of 58 children with a pediatric inflammatory multisystem syndrome temporally associated with SARS-CoV-2. JAMA. (2020) 324:259–69. 10.1001/jama.2020.10369 32511692 PMC7281356

[9] European Centre for Disease Prevention and Control. Week 23, 2021. Available online at: https://covid19-country-overviews.ecdc.europa.eu/ (accessed Jun 18, 2021)

[10] Centers for Disease Control and Prevention. Coronavirus disease 2019 in children — United States, February 12–April 2, 2020. MMWR Morb Mortal Wkly Rep. (2020) 69:422–6. 10.15585/mmwr.mm6914e4 32271728 PMC7147903

[11] Byun WS Heo SW Jo G Kim JW Kim S Lee S. Is coronavirus disease (COVID-19) seasonal? A critical analysis of empirical and epidemiological studies at global and local scales. Environ Res. (2021) 196:110972. 10.1016/j.envres.2021.110972 33705770 PMC7941024

[12] Leeb RT. COVID-19 trends among school-aged children — United States, March 1–September 19, 2020. MMWR Morb Mortal Wkly Rep. (2020) 69:1410-1415. 10.15585/mmwr.mm6939e2 33001869 PMC7537558

[13] Goldstein E Lipsitch M Cevik M. On the effect of age on the transmission of SARS-CoV-2 in households, schools, and the community. J Infect Dis. (2020) 223:362–9. 10.1093/infdis/jiaa691 33119738 PMC7665686

[14] Garazzino S Montagnani C Donà D Meini A Felici E Vergine G. Multicentre Italian study of SARS-CoV-2 infection in children and adolescents, preliminary data as at 10 April 2020. Euro Surveill. (2020) 25(18):2000600. 10.2807/1560-7917.ES.2020.25.18.2000600 32400362 PMC7219028

[15] European Centre for Disease Prevention and Control. Case Definition For Coronavirus Disease 2019 (COVID-19), as of 3 December 2020. Available online at: https://www.ecdc.europa.eu/en/covid-19/surveillance/case-definition (accessed Jun 18, 2021)

[16] Cui X Zhao Z Zhang T Guo W Guo W Zheng J. A systematic review and meta-analysis of children with coronavirus disease 2019 (COVID-19). J Med Virol. (2021) 93:1057–69. 10.1002/jmv.26398 32761898 PMC7436402

[17] Yasuhara J Kuno T Takagi H Sumitomo N. Clinical characteristics of COVID-19 in children: a systematic review. Pediatr Pulmonol. (2020) 55:2565–75. 10.1002/ppul.24991 32725955

[18] Kharoud HK Asim R Siegel L Chahal L Singh GD. Review of clinical characteristics and laboratory findings of COVID-19 in children-systematic review and meta-analysis. medRxiv. (2020) 10.1101/2020.09.23.20200410 (Accessed Jun 18, 2021) 32995815 PMC7523156

[19] Nacionalinis visuomenes sveikatos centras prie Sveikatos apsaugos ministerijos. Available online at: https://nvsc.lrv.lt/ (accessed Jun 18, 2021)

[20] Adlhoch C Mook P Lamb F Ferland L Melidou A Amato-Gauci AJ. Very little influenza in the WHO European region during the 2020/21 season, weeks 40 2020 to 8 2021. Euro Surveill. (2021) 26(11):2100221. 10.2807/1560-7917.ES.2021.26.11.2100221 33739256 PMC7976381

