# Supplementary figures and images for: Screening for Coronavirus Disease 2019 (COVID-19) at the Pediatric Emergency Department During Different Pandemic Phases
Source: Front Pediatr. 2021 Nov 5;9:749641. doi: 10.3389/fped.2021.749641 (PMC8603807; doi:10.3389/fped.2021.749641)

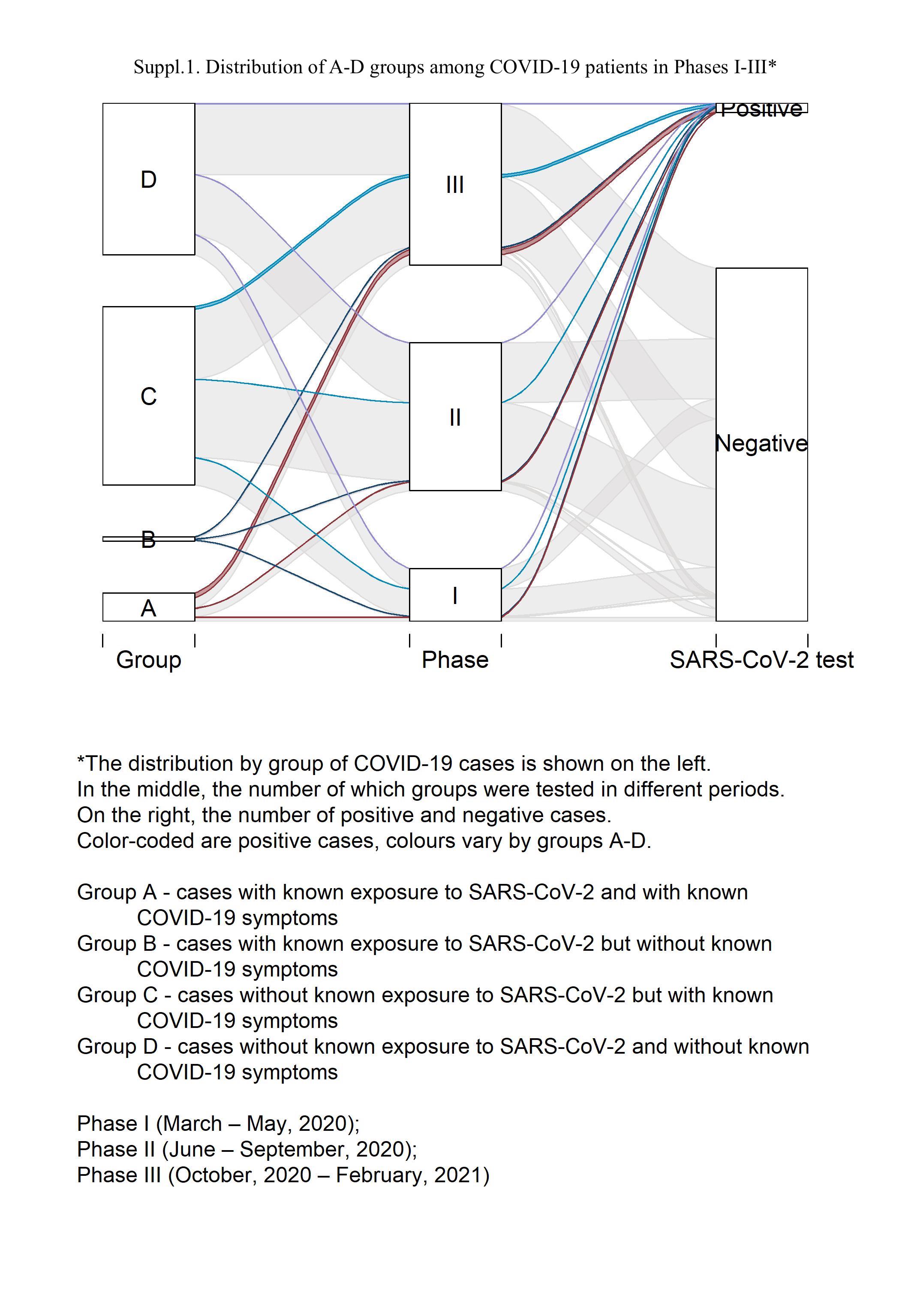

Supplement: Supplementary file 1 [file Image_1.JPEG]

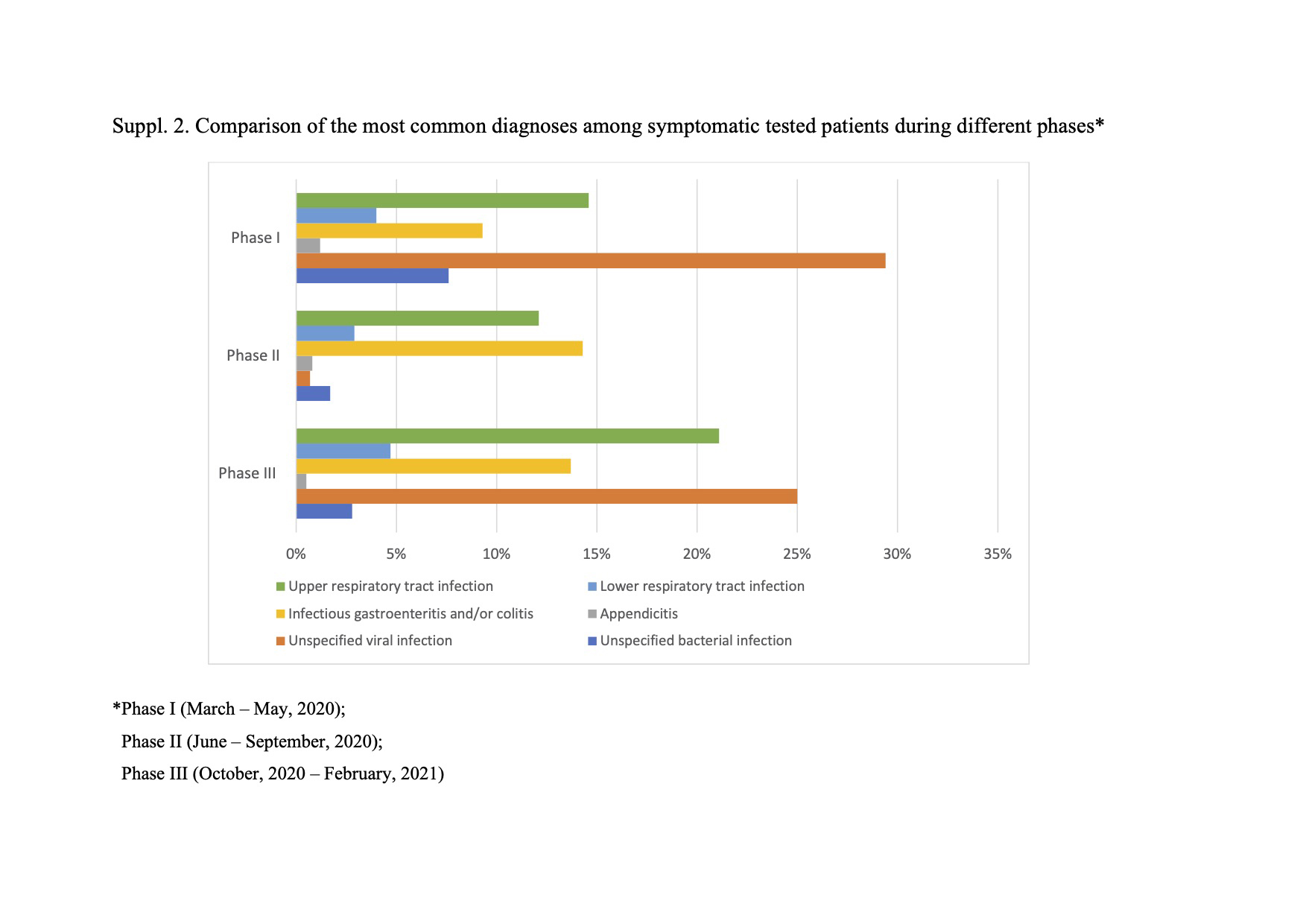

Supplement: Supplementary file 2 [file Image_2.JPEG]
